# Comparison of Mesothelial and Anti-mesothelial Surfaces of Tunica Vaginalis Flap as an Intermediate Layer in Urethroplasty: An Experimental Histopathological Study

**DOI:** 10.5152/tud.2026.25120

**Published:** 2026-08-14

**Authors:** Mehmet Şentürk, Hasan Deliağa, Esra Özçakır, Şule Bakır, Mete Kaya

**Affiliations:** 1 Department of Pediatric Surgery, Ministry of Health Bursa City Hospital, Bursa, Türkiye; 2 Department of Pediatric Urology, Bursa Yüksek İhtisas Education and Research Hospital, University of Health Sciences, Bursa, Türkiye; 3 Department of Pediatric Surgery, Bursa Yüksek İhtisas Education and Research Hospital, University of Health Sciences, Bursa, Türkiye; 4 Department of Pathology, Bursa Yüksek İhtisas Education and Research Hospital, University of Health Sciences, Bursa, Türkiye

**Keywords:** Urethra, urethroplasty, tunica vaginalis flap, wound healing, hypospadias

## Abstract

**Objective:**

Intermediate layers are commonly used to support the suture line in urethroplasty, with tunica vaginalis (TV) being one such option. However, it remains unclear which surface of the TV flap (TVF) is optimal for placement over the urethra. This study aimed to evaluate the effect of different TVF surfaces on urethral healing in an experimental model.

**Methods:**

Urethroplasty was performed on 24 adult male Wistar-albino rats, which were randomly assigned to 3 groups: control (urethroplasty only), mesothelial (mesothelial surface of TVF over urethra), and anti-mesothelial (anti-mesothelial surface over urethra). On postoperative day 21, urethral tissues were harvested and analyzed histopathologically using hematoxylin–eosin, Masson’s trichrome, and anti-alpha smooth muscle actin staining to assess inflammation, fibrosis, and neovascularization.

**Results:**

Inflammation scores did not differ significantly among the 3 groups (*P* = .163). Fibrosis scores differed significantly overall (*P* = .030). Pairwise comparisons demonstrated significantly higher fibrosis scores in the anti-mesothelial group than in the control group (*P* = .006) and the mesothelial group (*P* = .027). Fibrosis scores were also significantly higher in the mesothelial group than in the control group (*P* = .025). Neovascularization was significantly greater in the anti-mesothelial group than in the mesothelial group (*P* = .046). Neovascularization was not evaluated in the control group because no interposed tissue was used during urethroplasty.

**Conclusion:**

The orientation of the TVF may influence histopathological healing following experimental urethroplasty. While flap orientation was not associated with significant differences in inflammation, the anti-mesothelial orientation was associated with greater fibrosis and increased neovascularization than the mesothelial orientation. These findings should be interpreted cautiously because they are based on histopathological outcomes in an experimental model.

**Main Points**

- This randomized experimental study evaluated the histopathological effects of mesothelial and anti-mesothelial orientation of the tunica vaginalis flap used as an intermediate layer following urethroplasty in a rat urethral defect model.
- Overall fibrosis scores differed significantly among the groups (P = .030). Pairwise comparisons demonstrated significantly greater fibrosis in the anti-mesothelial group than in the control (P = .006) and mesothelial groups (P = .027).
- Although inflammation scores did not differ significantly among the groups, neovascularization was greater in the anti-mesothelial group than in the mesothelial group (P = .046).
- The orientation of the tunica vaginalis flap may influence histopathological wound healing after experimental urethroplasty. However, these findings are based on histopathological surrogate markers in an experimental model and require confirmation in studies evaluating functional and clinical outcomes.

## Introduction

Although different urethroplasty repair methods have been described for congenital and acquired urethral pathologies, many complications are frequently encountered. The frequency and association of complications, such as recurrent strictures, fistulas, or wound-related problems after urethroplasty, are uncertain and highly variable, with rates ranging from 1% to 70%.^1-4^

In order to prevent complications, many surgical techniques and additional procedures have been described. One of these is covering the urethra using well-vascularized tissues, such as subcutaneous tissue, dartos fascia, tunica vaginalis (TV), or corpus spongiosum, which has been shown to reduce complication rates, especially fistula formation.^5,6^

Since TV is close to the penis, it is used as a graft or flap material in penile and urethral surgeries.^7,8^ The main advantage of the described TV flap (TVF) is that it provides good intrinsic vascular supply from cremasteric vessels that do not rely on scarred local tissue.

It is known that while one surface of the TV is covered with mesothelium, the other surface contains connective tissue. Mesothelial cells form a monolayer that covers the entire surface of the pleural, pericardial, and peritoneal cavities. These cells possess microvilli on their surface, providing a protective barrier and facilitating frictionless movement between opposing surfaces. Additionally, mesothelial cells play a key role in the absorption of solutes and fluids between the vascular system and body cavities. Their crucial functions include fibrin deposit lysis, tissue repair, initiation and resolution of inflammation, and protection against invading microorganisms and tumor dissemination.^9^

To date, no study has been published showing whether it is more appropriate to place the inner mesothelial surface or the outer connective tissue of the TVF on the urethra in urethroplasty. In our experimental study based on this histopathological research, the effects of mesothelial and anti-mesothelial surfaces of TVF on wound healing in the urethroplasty model were investigated.

## Material and Methods

Twenty-four male Wistar albino rats (300 ± 60 g body weight) were used for this study and randomly divided into 3 groups of 8 animals each: control, mesothelial, and anti-mesothelial. Because of the exploratory nature of this experimental study, no a priori power analysis was performed. The sample size was based on previous comparable studies and the principle of minimizing animal use. The experimental protocol was approved by the Ethics Committee of University of Uludağ (Approval No. 2021-11/09, Date: September 5, 2021). All animal procedures were conducted in accordance with institutional guidelines and the National Institutes of Health Guide for the Care and Use of Laboratory Animals. The experimental study was conducted between March 19, 2022, and May 15, 2022. This study used ChatGPT (OpenAI) to improve the English language, grammar, and clarity of the manuscript during its preparation. The AI tool was also used to assist in editing the responses to the reviewers. All scientific content, data analysis, interpretation of the results, and final revisions were performed and verified by the authors, who take full responsibility for the content of the manuscript.

### Procedures

A single dose of ceftriaxone (Rocephin® IM 1 g; Roche, Istanbul, Turkey) at 50 mg/kg was administered to each group of animals intramuscularly. Following induction of anesthesia with sevoflurane (Sevorane®, Abbott, Wiesbaden, Germany) inhalation, the rats were positioned supine. The surgical site was prepared with topical povidone-iodine antiseptic and covered with sterile drapes. The penis was exposed, and a metal stylet was inserted into the urethra. Under a surgical microscope (Zeiss OPMI 1-FC®, Carl Zeiss Meditec AG, Jena, Germany), a midline ventral skin incision was made at the penile root using a microscalpel. A 2-mm urethral incision was then performed to generate a urethral defect (Figure 1A and 1B). The defect was then repaired in a full-thickness manner using a 7/0 polyglactin 910 (Vicryl®, Ethicon, Inc., Somerville, NJ, USA) suture at the end of the procedure.

**Figure 1.**
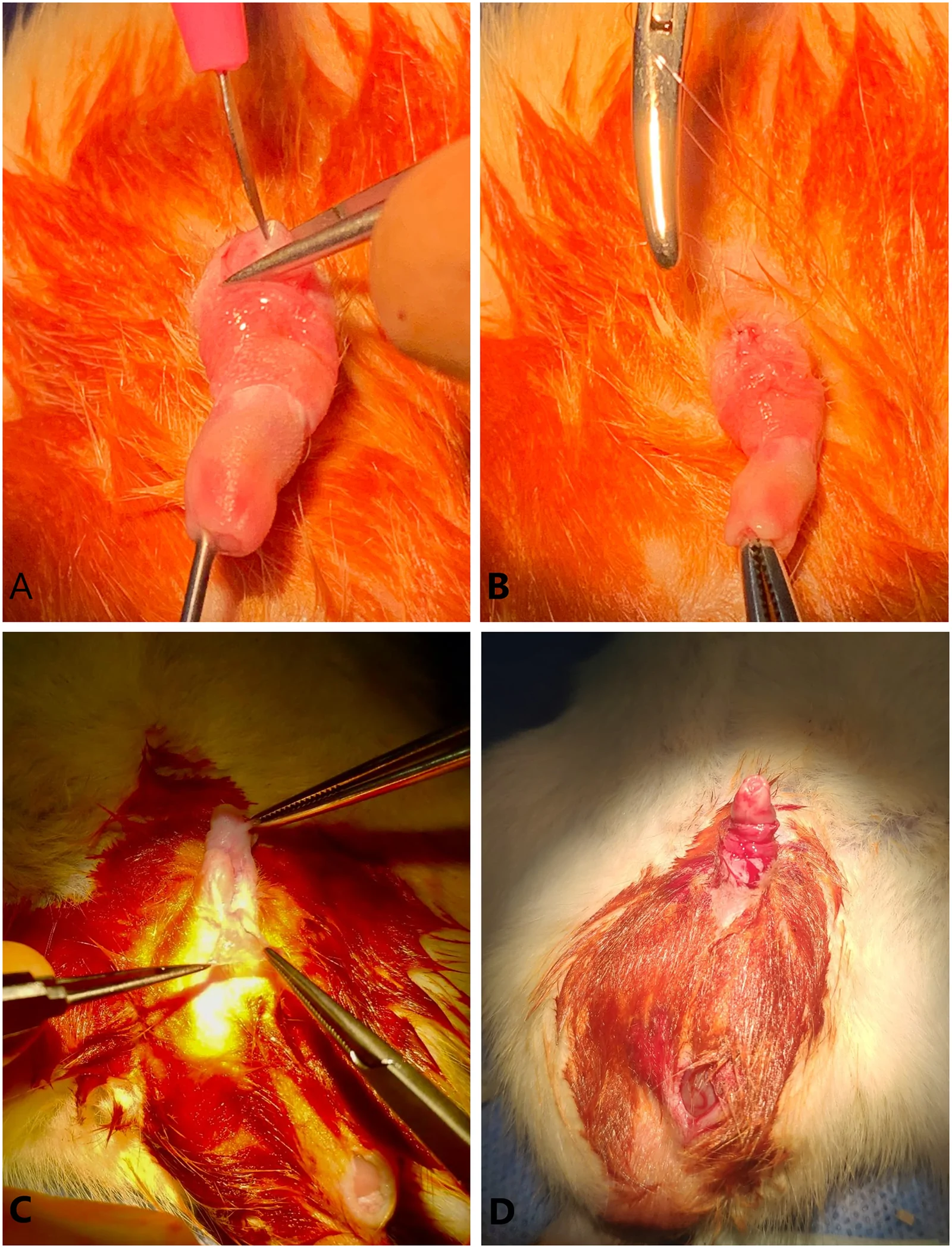
Intraoperative views of TVF application in the experimental urethroplasty model. (A) Creation of a standardized urethral defect via midline ventral incision and probing. (B) Primary repair of the defect with full-thickness sutures to complete urethroplasty. (C) Transposition of the TVF, harvested via scrotal incision, through a subcutaneous tunnel onto the urethral repair site. (D) Final view after coverage with TVF and closure of the penile skin. Abbreviation: TVF, tunica vaginalis flap.

TVF was prepared to be used in the experimental groups, following the method described by Theodorescu et al^10^ The left testis was delivered through a longitudinal scrotal incision. A longitudinal flap was prepared with a U-shaped incision from the parietal TV. The TVF was then tunneled subcutaneously into the previously created ventral defect of the penis. This subcutaneous tunnel was extensively dissected to prevent compression of the vascular pedicle. Finally, the vascularized TVF was positioned without tension over the repaired corpus spongiosum, covering the reconstructed urethra (Figure 1C and 1D).

In the control group, the skin was closed with the same suture material without using an intermediate layer. In the mesothelial group, the TVF was rotated 180° in the tunnel and its visceral surface was laid on the urethra, and it was sutured to the corpus cavernosum. In the anti-mesothelial group, the TVF was passed through the tunnel without being rotated and the anti-mesothelial surface was laid on the urethra (Figure 2). The skin was then closed continuously and in full thickness with the same suture.

**Figure 2.**
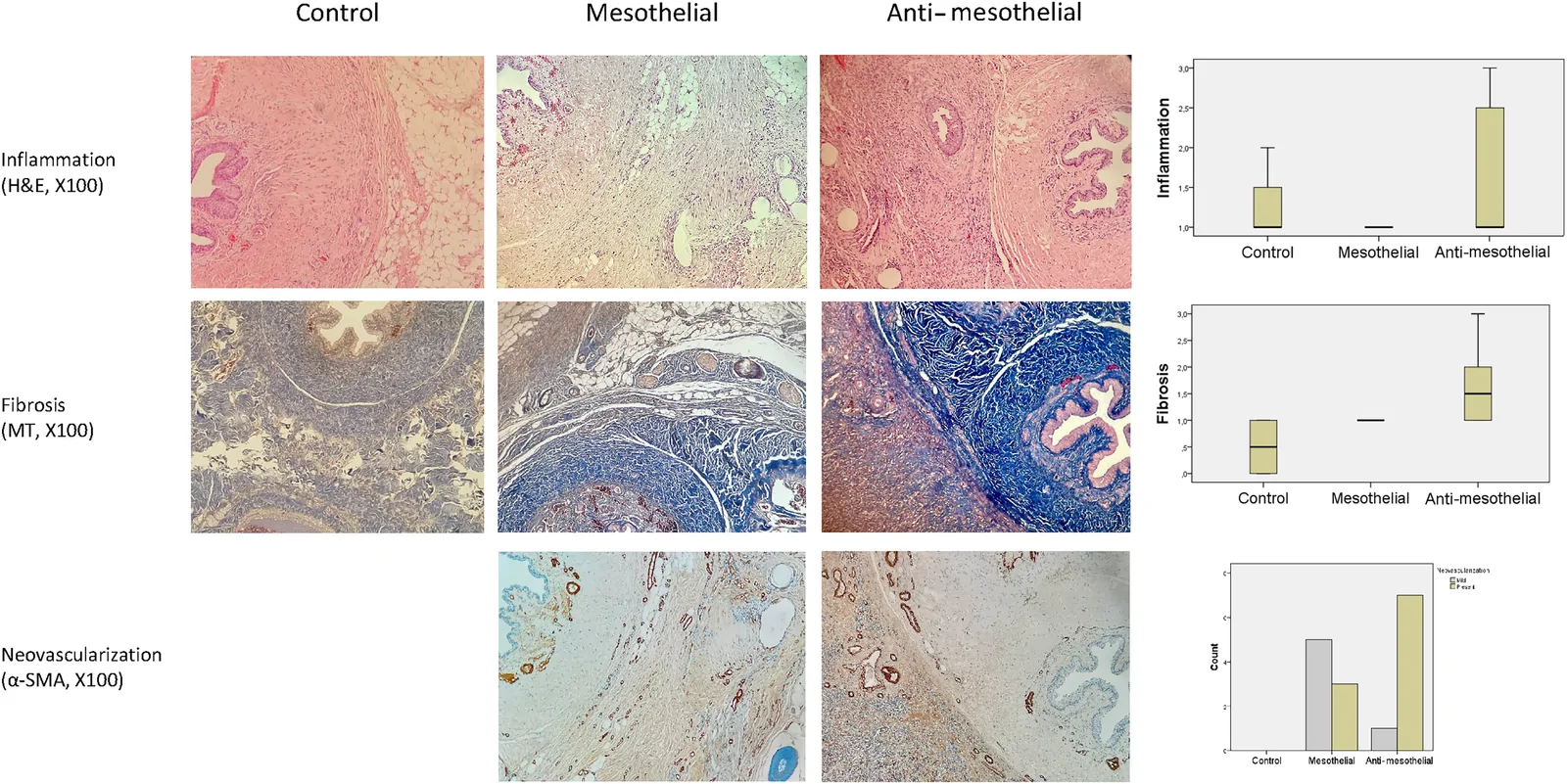
H&E, MST, and α-SMA staining of the urethra of rats. Upper row: Increased inflammatory reaction with epithelial thickening and macrophage accumulation in a rat of mesothelial group (H&E stain, 100×), and box plots for histopathologic scores of inflammatory reactions. Middle row: The patent lumens had normal urothelial lining with different degrees of fibrosis between the urethral and skin (MST stain, 100×), and box plots for fibrosis scores of the groups. Lower row: Expression of α-SMA in rats according to neovascularization (α-SMA stain, 100×), and box plots for degree of neovascularization. Abbreviations: α-SMA, alpha smooth muscle actin; H&E, hematoxylin and eosin; MST, Masson’s trichrome.

### Postoperative Care

The animals were recovered and returned to care facility. All animals were kept on a 12-hour light:12-hour darkness cycle at 22°C–24°C with free access to food and fasted for 24 hours before the operation, but were allowed free access to water. No postoperative analgesia was administered, in accordance with the protocol approved by the Institutional Animal Care and Use Committee. They were examined daily to monitor wound healing. All animals were euthanized by cervical dislocation following 21 days postoperative observation. After 21 days of postoperative observation, all animals were deeply anesthetized with sevoflurane inhalation and euthanized by cervical dislocation. The penis was then removed en bloc for histopathological examination.

### Histopathology and Evaluation

The resected penile segments were fixed in formaldehyde (10%) solution. All samples were embedded in paraffin. Tissues were sectioned in 4-µm pieces and stained with hematoxylin and eosin, Masson’s trichrome (MST), and monoclonal anti-alpha smooth muscle actin (α-SMA) primary antibodies. Microscopic analysis of stained specimens was performed by a single experienced pathologist under a light microscope (Olympus® BX51, Tokyo, Japan). Histological features, including the extent and nature of inflammatory cell infiltration, the extent of fibrosis with collagen deposition, and neovascularization with the formation of a smooth muscle layer and rich vessels, were systematically evaluated in each animal.

The samples were examined according to the histopathological criteria described by Calado et al^11^ Inflammation was graded on a scale from 0 to 4 based on the extent of inflammatory cell infiltration. The presence of polymorphonuclear cells, monocytes, lymphocytes, macrophages, and multinucleated giant cells was considered during the histopathological assessment of inflammation.

Fibrosis assessment was performed using the method described by Atıcı et al,^12^ with area measurements performed on 10 independent microscopic fields (magnification 200×, MST). These were distributed equally throughout the proximal, distal, and central regions of the original surgical sites to determine the percentage of tissue area occupied by MST+ tissue relative to the total tissue area examined. Fibrosis was assessed as 0 = none, 1+ = tissues with <10% fibrosis (mild), 2+ = tissues with 10%–49% fibrosis (moderate), and 3+ = tissues with ≥50% fibrosis (severe). Immunohistochemical staining for α-SMA was performed to evaluate α-SMA expression. The degree of immunoexpression was determined according to the staining intensity and the number of positively stained cells. Immunoreactivity was assessed semiquantitatively as absent, mild, moderate, or high based on the intensity of the staining.^13^ Ten high-power fields (400×) were evaluated for each specimen, and the staining intensity was recorded.

### Statistical Analysis

Statistical analysis was performed using IBM SPSS Statistics for Windows, version 22.0 (IBM Corp., Armonk, NY, USA). Data are presented as frequencies and percentages. Since the data were ordinal and non-normally distributed, non-parametric tests were used for comparisons. Overall differences among the 3 groups were evaluated using the Kruskal–Wallis test. When a significant overall difference was detected, pairwise comparisons were made using the Mann–Whitney *U* test. To control for type I error due to multiple pairwise comparisons, the Bonferroni correction was applied, and a corrected significance threshold of *P* < .0167 (0.05/3) was used. When expected cell frequencies were below 5 in contingency tables, Fisher’s exact test was used. Exact *P* values are reported where applicable.

## Results

Until postoperative day 21, wound healing was uneventful in all rats except for 2 in the anti-mesothelial group, which developed surgical site infection and granulation tissue. Neither rat exhibited any urinary dysfunction. Both animals completed the study and were included in the histopathological and statistical analyses. Examination of hematoxylin and eosin–stained sections revealed epithelial thickening in 2 rats and excessive macrophage accumulation in 2 rats of the anti-mesothelial group, representing a subacute inflammatory reaction. In the mesothelial group, giant cell formation was observed in 1 rat and minimal epithelial thickening in another, with no evidence of a foreign body reaction. Histopathological scores are summarized in Table 1.

**Table 1.** Analysis of Histopathologic Evaluation Following Urethroplasty With Tunica Vaginalis Flap Covering

Histopathological Criteria	Control (*n*)	Mesothelial (*n*)	Anti-mesothelial (*n*)	Overall *P* Value
Inflammation				
Score 0	—	—	—	**.163** ^a^
Score 1	6 (75%)	8 (100%)	5 (63%)	
Score 2	2 (25%)		1 (12%)	
Score 3	—		2 (25%)	
Score 4	—	—	—	
Fibrosis				
Score 0	4 (50%)	—		**.03** ^a^
Score 1+	4 (50%)	8 (100%)	5 (63%)	
Score 2+	—	—	2 (25%)	
Score 3+	—		1 (12%)	
Neovascularization				
Absent	—			**.046** ^b^
Mild	—	8 (100%)	1 (12%)	
Present	—	—	7 (88%)	

a Kruskal–Wallis test.

b Mann–Whitney *U* test (comparison between mesothelial and anti-mesothelial groups only).

Mean inflammation scores were mild in both the control and mesothelial groups. In the anti-mesothelial group, most rats exhibited mild inflammation, whereas 3 rats showed moderate or severe inflammation (Figure 2). The Kruskal–Wallis test showed no statistically significant difference in inflammation scores among the 3 groups (*P* = .163). Pairwise comparisons also demonstrated no statistically significant differences between the control and anti-mesothelial groups (*P* = .441), the control and mesothelial groups (*P* = .143), or the mesothelial and anti-mesothelial groups (*P* = .064).

In MST staining, half of the control group showed no fibrosis, while the other half showed mild fibrosis. In the anti-mesothelial group, mild fibrosis (1+) was observed in 5 rats (63%), moderate fibrosis (2+) in 2 rats (25%), and severe fibrosis (3+) in 1 rat (12%). In the mesothelial group, mild fibrosis was observed in all rats (Figure 2). The Kruskal–Wallis test demonstrated an overall difference in fibrosis scores among the 3 groups (*P* = .03). Pairwise comparisons with Bonferroni correction showed significantly higher fibrosis scores in the anti-mesothelial group than in the control group (*P* = .006) and in the mesothelial group (*P* = .027). No statistically significant difference was demonstrated between the control and mesothelial groups (*P* = .025).

When neovascularization was examined with α-SMA immunohistochemical staining, it was seen as mild or obvious in the mesothelial group and very obvious in the anti-mesothelial group (Figure 2). The differences in neovascularization between the groups were significantly increased in the anti-mesothelial group compared with the mesothelial group (*P* = .046). Neovascularization was not assessed in the control group, as no interposed tissue was introduced in this group during the urethroplasty procedure.

## Discussion

Although advancements in surgical techniques and the development of novel biomaterials have significantly improved the success rates of urethroplasty, the procedure still carries a considerable risk of postoperative complications, most notably urethrocutaneous fistula and stricture formation.^5^ To minimize postoperative complications, vascularized tissues, such as subcutaneous, dartos, and spongious, are frequently interposed between the neourethra and the overlying skin as a second protective layer. Although a variety of tissues have been utilized for this purpose to improve surgical outcomes, their use may be associated with complications such as skin necrosis, penile torsion, or chordee. In some cases, these tissues may not be available, and in such instances, a TVF is employed as an alternative intermediate layer.^14^ The TVF has previously been utilized as a graft in the treatment of Peyronie’s disease and for closing defects in the tunica albuginea following chordee repair. More recently, it has been employed both as a urethral substitute and as an intermediate layer in urethroplasty. Its use enhances vascularity, provides an additional protective layer, and helps prevent the overlapping of suture lines.^8,10,15,16^ Owing to its unique anatomical origin and histological structure, the TV serves as an ideal candidate for use as an intermediate layer in urethral surgery. Anatomically, the TV derives from the abdominal peritoneum, continues along the inguinal canal, covers the inner part of the scrotum and surrounds the ducts associated with the testicles and spermatic cord.

To date, there has been no clear consensus regarding which surface of the TVF should face the neourethra to achieve optimal tissue coverage. Only a limited number of studies have described the orientation of the flap in detail. In a study by Tavakkoli Tabassi and Mohammadi,^17^ the serosal (mesothelial) surface of the TVF was placed against the ventral aspect of the urethra. Among the 10 patients in their series, complications were reported in 34%, including urethrocutaneous fistula (10%), wound infection (14%), and meatal stenosis (10%). In their study, Kumar et al^18^ utilized the mesothelial surface of the pedicled TVF to wrap the repaired urethra dorsally during bulboprostatic anastomotic urethroplasty in 4 patients. They reported favorable outcomes during the postoperative follow-up period.

In the study by Chatterjee et al,^19^ TVF was used following urethroplasty in patients with hypospadias. Although the authors did not explicitly state which surface of the flap was applied to the neourethra, the accompanying illustrations suggest that the mesothelial surface was placed in contact with the urethra. In contrast, Pescheloche et al^20^ applied the connective tissue (anti-mesothelial) side of the TVF onto the neourethra, positioning the testicular (mesothelial) surface anteriorly, beneath the skin, during urethrocutaneous fistula repair. This approach yielded highly successful outcomes, with a reported 100% success rate. Similarly, Routh et al^21^ used the anti-mesothelial surface of the TVF over the repaired urethra by creating a subcutaneous tunnel, achieving favorable results in urethrocutaneous fistula cases. In several other studies, no clear description or visual representation was provided regarding which surface of the flap was placed against the urethra. In the present experimental study, urethroplasty was performed using both orientations of the TVF to evaluate their application. Consistent with clinical practice, the flap was delivered into the surgical field through a skin tunnel. When the anti-mesothelial side was used, it was applied directly onto the urethra without torsion. However, the application of the mesothelial side required a 180° torsion of the flap within the tunnel. A detailed review of clinical studies suggests that when the mesothelial surface is used, the flap is intentionally twisted to orient this surface toward the neourethra. Clinical studies have reported successful outcomes with the use of both the mesothelial and anti-mesothelial surfaces of the TVF in preventing urethroplasty-related complications. However, no clear consensus or recommendation exists regarding which surface should be oriented toward the neourethra. To position the mesothelial surface of the TVF directly against the urethral repair, a 180° rotation of the flap was required. This resulted in a pedicle torsion that was not present in the anti-mesothelial group. However, no alternative surgical approach was available to achieve direct apposition of the mesothelial surface while preserving flap vascularity. Therefore, pedicle torsion should be considered an inherent technical aspect of this surgical technique rather than an independent experimental variable. Nevertheless, if pedicle torsion exerts any biological effect, it may represent the principal potential disadvantage of positioning the mesothelial surface directly over the urethral repair.

Histologically, the inner surface of the TV consists of a smooth mesothelial lining composed of simple squamous epithelial cells, forming a serous membrane that facilitates frictionless movement of the testicular structures.^22^ The TVF, with its serosal surface, serves as a dependable source of mesothelial cells that is both readily accessible and associated with a low risk of intra-abdominal morbidity. The serosal (mesothelial) surface of the TVF is considered favorable for use in urethral reconstruction.

In the present study, the placement of the mesothelial surface against the urethra led to improved wound healing, as evidenced by decreased inflammation and fibrosis on histopathological evaluation. Although inflammation is an essential component of normal wound healing and contributes to tissue repair, sealing, and host defense during the early phase, excessive or prolonged inflammation may promote fibroblast activation, extracellular matrix deposition, and subsequent fibrosis.^11^ Therefore, the lower degree of inflammation observed in our study likely reflects a more regulated healing response rather than suppression of physiological inflammation. Consistent with the biological properties of mesothelial cells, the limited inflammation and reduced fibrosis observed in the present study may be explained by enhanced fibrinolysis and efficient regulation of the inflammatory response.^9^ Upon injury to serosal surfaces, mesothelial cells desquamate, leading to inflammation and fibrin meshwork formation over the exposed area. Subsequently, islands of mesothelial cells regenerate and re-epithelialize the injured surface, with increasing fibrinolytic activity as the cells proliferate. Insufficient fibrinolytic activity, however, results in persistent fibrin deposits, causing fibrous adhesions between opposing serosal surfaces.^23,24^ In the present study, the absence of a serosal cavity into which shed mesothelial cells could disperse may have contributed to the limited inflammation and reduced fibrosis observed, possibly through enhanced fibrinolysis and more efficient resolution of the inflammatory response. Our findings support our original hypothesis that the mesothelial surface of the TVF may facilitate favorable wound healing. Although fibrosis was observed in both TVF groups compared with the control group, fibrosis was significantly less pronounced when the mesothelial surface faced the urethra than when the anti-mesothelial surface was applied. These findings partially support our original hypothesis by suggesting that preservation of the mesothelial orientation may attenuate the fibrotic response, although the interposition of the flap itself was associated with greater fibrosis than urethroplasty alone. Nevertheless, our findings suggest that this tissue can effectively function as an intermediate layer even when the serosal surface is not maintained in its natural anatomical environment.

Mesothelial cells, which are transitional mesodermal cells, possess the potential to differentiate into endothelial cells and have been demonstrated in experimental models to induce neovascularization.^25^ This phenomenon may be attributed to the tissue support provided by the well-vascularized TVF positioned between the skin and urethra. The increased neovascularization observed in the anti-mesothelial group may be attributed to an inflammatory response triggered by the interaction between the mesothelial layer and the more vascularized subcutaneous tissue. In contrast, the lower incidence of neovascularization in the mesothelial group may reflect the reduced inflammatory response elicited by this surface. However, the generally low levels of neovascularization in both groups are likely attributable to the early phase of wound healing assessed in this study.

The limitations of this study include the relatively small sample size and the lack of more detailed histopathological analyses. Employing advanced ultrastructural techniques to evaluate inflammation and fibrosis may provide more definitive guidance regarding the optimal orientation of the TVF. Histopathological evaluation was also performed by a single non-blinded pathologist, which may have introduced observer bias. Another limitation of the present study is that neovascularization was evaluated using α-SMA immunostaining. Because α-SMA is not specific for endothelial cells and also labels vascular smooth muscle cells, pericytes, and myofibroblasts, the observed staining may not exclusively represent newly formed blood vessels. Therefore, the neovascularization findings should be interpreted with caution. Future studies using endothelial-specific markers, such as CD31 or CD34, and microvessel density analysis may provide a more accurate assessment of angiogenesis. Furthermore, the experimental model represents primary repair of a urethral defect rather than hypospadias repair, and anatomical differences between the rat urethra and the human urethra may further limit the direct clinical applicability of the findings. Currently, the implications of our findings remain speculative due to limited available data.

In conclusion, coverage of urethroplasty with TVF remains a well-established technique because of its rich vascularity and ease of application as an intermediate layer. In the present experimental study, placement of the mesothelial surface against the urethral repair was associated with reduced inflammation and fibrosis at postoperative day 21 compared with the anti-mesothelial orientation. These findings suggest that the orientation of the TVF may influence histological wound healing. However, because this study evaluated only histopathological surrogate markers at a single time point and did not assess clinical outcomes, such as fistula formation, urethral stricture, or urethral patency, these findings should be interpreted with caution. Further well-designed experimental and clinical studies are required to determine whether these histological differences translate into improved clinical outcomes and to establish recommendations regarding the optimal orientation of the TVF.

## Data Availability

The data that support the findings of this study are available on request from the corresponding author.
